# The validity of the Child Health Utility instrument (CHU9D) as a routine outcome measure for use in child and adolescent mental health services

**DOI:** 10.1186/s12955-015-0218-4

**Published:** 2015-02-18

**Authors:** Gareth Furber, Leonie Segal

**Affiliations:** Health Economics and Social Policy Group, School of Population Health, University of South Australia, Adelaide, Australia

**Keywords:** Health related quality of life, Child and adolescent mental health services, Validation

## Abstract

**Background:**

Few cost-utility studies of child and adolescent mental health services (CAMHS) use quality adjusted life years (a combination of utility weights and time in health state) as the outcome to enable comparison across disparate programs and modalities. Part of the solution to this problem involves embedding preference-based health-related quality of life (PBHRQOL) utility instruments, which generate utility weights, in clinical practice and research. The Child Health Utility (CHU9D) is a generic PBHRQOL instrument developed specifically for use in young people. The purpose of this study was to assess the suitability of the CHU9D as a routine outcome measure in CAMHS clinical practice.

**Methods:**

Two hundred caregivers of children receiving community mental health services completed the CHU9D alongside a standardised child and adolescent mental health measure (the Strengths and Difficulties Questionnaire – SDQ) during a telephone interview. We investigated face validity, practicality, internal consistency, and convergent validity of the CHU9D. In addition, we compared the utility weights obtained in this group with utility weights from other studies of child and adolescent mental health populations.

**Results:**

Participants found the CHU9D easy and quick to complete. It demonstrated acceptable internal consistency, and correlated moderately with the SDQ. It was able to discriminate between children in the abnormal range and those in the non-clinical/borderline range as measured by the SDQ. Three CHU9D items without corollaries in the SDQ (sleep, schoolwork, daily routine) were found to be significant predictors of the SDQ total score and may be useful clinical metrics. The mean utility weight of this sample was comparable with clinical subsamples from other CHU9D studies, but was significantly higher than mean utility weights noted in other child and adolescent mental health samples.

**Conclusions:**

Initial validation suggests further investigation of the CHU9D as a routine outcome measure in CAMHS is warranted. Further investigation should explore test-retest reliability, sensitivity to change, concordance between caregiver and child-completed forms, and the calibration of the utility weights. Differences between utility weights generated by the CHU9D and other utility instruments in this population should be further examined by administering a range of PBHRQOL instruments concurrently in a mental health group.

## Background

Routine outcome measurement in mental health services involves the use of generic measures to assess change in consumers’ functioning, performance or participation over time [1,2]. Routine outcome measurement serves multiple purposes. At the consumer level, measures can be used to monitor therapy progress and foster dialogue about treatment goals. Clinicians and supervisors can use measures for reflective practice, to choose appropriate treatments, to determine eligibility for treatment, and for discharge planning. Services can use aggregated data from measures for quality improvement activities and to foster evidence-based practice. Finally, funders and policy makers can use data aggregated from services to make decisions about resource allocation [1-4].

In Australian child and adolescent mental health services (CAMHS), 2 instruments are commonly used to track outcomes during a clients’ episode of care. The Health of the Nation Outcome Scales for Children and Adolescents (HONOSCA) is a clinician-completed 15-item measure of a child’s symptoms and social and physical functioning [5]. The Strengths and Difficulties Questionnaire (SDQ) is a caregiver- or child/adolescent-completed 25-item brief behavioural screening questionnaire [6]. The 2 measures are complementary. The HONOSCA supports clinicians in rating a child’s functioning across important diagnostic and functional domains. The HONOSCA can be used at the individual level to guide treatment decisions, but also at the organisational level to profile the population receiving care. The SDQ provides an opportunity for young people or caregivers to rate emotional and behavioural symptoms to track progress during intervention. In both cases, when used at more than 2 time points during an episode of care, the instruments can be used to monitor the outcomes of intervention.

In a previous paper we recommended that CAMHS consider integrating preference-based health-related quality of life (PBHRQOL) instruments into routine outcome measurement practice [7]. Health-related quality of life refers to an individual’s perception of their physical and mental health and thus PBHRQOL instruments are used to rate an individual’s functioning across a range of domains (e.g. Independent Living, Happiness, Mental Health, Coping, Relationships, Self Worth, Pain, Sensation). In contrast to the SDQ and HONOSCA which are mental health focused, PBHRQOL instruments are commonly generic, assessing domains relevant to individuals with many types of illness.

PBHRQOL instruments are unique amongst standardised instruments in that they generate utility weights. Utility weights have significant value in health policy, as they are used to calculate what is known as ‘quality-adjusted life-years’ (QALY), a measure of health used in the evaluation of health-related interventions. The value of the QALY is that it can capture both quality of life, and life expectancy effects as a result of intervention, and its generic form facilitates comparison of the cost-effectiveness of health interventions from diverse areas. The QALY is now the standard outcome measure in health economic evaluation and used by key national health bodies such as the National Institute of Health and Care Excellence (NICE) in the UK, and the Pharmaceutical Benefits Advisory Committee (PBAC) and Medical Services Advisory Committee (MSAC) in Australia. This is an important point. In health, where there are limited budgets, those services/programs/interventions that can demonstrate their benefits using metrics employed by key policy advisory groups, increase their chances of funding. This is the primary logic behind our recommendation for these metrics to be embraced by mental health services, which compete, at least partially, with pharmaceuticals for funding. Pharmaceutical companies are well versed in using these metrics to show the benefits of their products.

Utility weights are calculated by applying a special algorithm or tariff to an individual’s responses on the PBHRQOL instrument. These tariffs are derived from a valuation process in which members of the general population rank between 0 (representing death) and 1 (representing full health), the different health states described by the instrument. In practice, ranking all the health states is impossible. A 9-item instrument like the CHU9D discussed in this paper, can generate almost 2,000,000 health states (9 items, 5 levels each - 5^9^). Health economists use specialised modelling to predict utility weights for all the different health states, by observing the population’s response to a subset of them. There are different valuation processes (e.g. standard gamble, time-trade off) and modelling methods [8]. As such, a single instrument may have different tariffs for different population groups that are used to generate utility weights. For example, the CHU9D has 2 tariffs— a UK Adult Tariff [9] and an Australian Adolescent Tariff [10].

There is a growing range of PBHRQOL instruments available for use with children and adolescents including the Health Utilities Index (HUI) [11], 16D and 17D [12,13], EQ-5D-Y [14], Adolescent Health Utility Measure (AHUM) [15] and the Assessment of Quality of Life – 8 Dimension (AQoL-8D) [16]. They range in size from the 5-item, 5-dimension EQ-5D-Y to the 35-item, 8-dimension AQoL-8D. They also range in scope from the AQoL-8D and AHUM which are suitable for use in adolescents to the HUI which can (with proxy measurement) be used in children as young as 5. A relatively new instrument called the Child Health Utility – 9D (CHU9D) has been the subject of a number of recent publications [17-25].

### The CHU9D

The CHU9D [17-20] was designed for use in children aged 7–11 years, but with interviewer assistance can be used in children as young as 6 [21], and research has demonstrated its validity in adolescents up to age 17 [22]. It consists of 9 items, each with 5 response categories (scored 1–5) that assess the child/adolescent’s functioning “today” across domains of worry, sadness, pain, tiredness, annoyance, school, sleep, daily routine and activities. The instrument is available in both self-report (completed by the child) and proxy-report (caregiver completed) forms.

The CHU9D was developed in response to a perceived paucity of paediatric preference-based measures for use in health care resource-allocation decision making [17]. The 9 domains of the questionnaire were identified from qualitative interviews with children aged 7 to 11 years, who described the areas of their life affected by their health conditions [18]. As with other PBHRQOL instruments, the CHU9D has undergone ‘valuation’ where the various health states described by the instrument (i.e. potential combination of scores across the different items) have been valued by the general public generating tariffs for calculating utility weights from an individual’s score [9]. In fact, 2 sets of preference weights (tariffs) are available. The first (UK Adult Tariff) was generated from health state valuation interviews with 300 members of the UK adult general population [9] using the standard gamble method. This tariff generates utility weights between .33 and 1. The second set (Australian Adolescent Tariff) was developed by Ratcliffe and colleagues [10], based on interviews with 590 Australian adolescents using profile case best-worst scaling (BWS) discrete-choice experiment (DCE) methods. It similarly generates values between .33 and 1, although demonstrates some significant differences in the valuation of some health states, particularly related to mental health attributes [22].

There are a number of features of the CHU9D that make it a potential candidate as a routine outcome measure in child and adolescent mental health. It was developed using research with children, is brief and simply worded, has a low response burden, is available in proxy and self-report forms, has been used in children and adolescents from 7–18 years old, uses a shortened reference time frame (“today”) suitable for repeated measurements, has a good representation of mental health related items (sad, worried, annoyed), and is impact rather than symptom focused, complementing existing measures. Previous validation studies with adolescents from the community have found the instrument to be well understood, to discriminate between individuals based on their self-reported health status and show expected correlations with other generic quality of life instruments [22,24]. A validation study with children aged 6–7 [21] showed they appeared to comprehend the questions when asked by an interviewer, but there was some doubt as to the reliability of their answers, given relatively low test-retest reliability. Validation studies with clinical populations have not been carried out to our knowledge.

Our own unpublished pilot testing of the CHU9D with children, caregivers and CAMHS providers indicated that children as young as 6 could complete the instrument with assistance, that caregivers found the instrument brief and simple to use, and providers felt the instrument provided a reasonable overview of the child’s functioning. There was also correlational evidence that a young person’s score on the CHU9D (either self-report or proxy) corresponded with their clinical severity as indicated by the service provider.

The purpose of this paper is to report on the findings of using the proxy version of the CHU9D alongside the widely-used Strengths and Difficulties Questionnaire (SDQ) with 200 caregivers of children receiving mental health services. As our aim was to determine whether the CHU9D would make a suitable instrument for use in CAMHS, we explored multiple aspects of its performance: face validity, practicality, internal consistency, and convergent validity. We also compared the utility weights obtained in this child and adolescent mental health population, with utility weights from other studies of child and adolescent mental health populations.

## Method

### Design

The study employed a cross-sectional telephone survey design, in which caregivers of children receiving services from a local child and adolescent mental health service were asked to complete the CHU9D and the SDQ in a single sitting. The study was approved by both Health Service (#384.11) and University ethics committees (#25739).

In this study, we sought to answer 5 questions about the CHU9D, relevant to its potential use as a routine outcome measure in CAMHS. These are summarised in Table 1.

**Table 1:** **Study research questions**

**Topic**	**Research question**	**Outcome if CHU9D is appropriate for use in CAMHS**
Face validity and practicality	1. Is the CHU9D simple to understand and complete?	CHU9D should be well understood and easily completed by participants.
Reliability	2. What is the internal consistency of the CHU9D?	Instrument should have acceptable to excellent internal consistency indicating the items are tapping into the same general construct (health-related quality of life).
Convergent validity	3. How do items and scores on the CHU9D correlate with items and scores from the Strengths and Difficulties Questionnaire (SDQ), a well-established and widely used routine outcome measure in child and adolescent mental health?	Items and scores on the CHU9D should demonstrate predictable and moderate to high correlations with items and scores on the SDQ, indicating they are tapping into the same broad construct (psychosocial functioning).
	4. Can the CHU9D discriminate between children in the clinical and non-clinical range on the SDQ?	Scores on the CHU9D should be able to discriminate between children at different mental health symptom severity levels.
Validity of utility weights	5. How do the utility weights from this child and adolescent mental health sample compare against utility weights obtained from other child and adolescent mental health samples?	The utility weights that the CHU9D generates for this mental health population should reflect utility weights obtained in other similar mental health populations.

### Participants

Participants were parents or other adult relatives of children aged 5–17 years (inclusive), who were registered as ‘current clients’ of a regional child and adolescent mental health service. ‘Current client’ status was defined as having an open episode of care and a recorded contact within the last 6 weeks. Excluded were caregivers who had no recorded telephone number, had specific “no contact” instructions in the electronic clinical record, were foster carers, or whose child was the subject of current guardianship or family court orders.

### Procedure

Potential participants were identified from the electronic clinical record of the CAMH service and placed on a list. The order of participants on the list was randomised before being provided to telephone interviewers. All listed participants were sent out introductory letters at least one week prior to being contacted by phone by interviewers. Where a participant was identified as having more than one child receiving CAMH services, a coin toss method was used to identify which child the participant would be asked to rate.

### Measures

A telephone survey was developed that consisted of the CHU9D, the SDQ and additional demographic, presenting issue, and service satisfaction questions. The order of presentation was the same for all participants. Child health status and emotional and behavioural health were assessed by proxy (i.e. by the child’s caregiver). Proxy outcome measurement is common practice both in CAMH services and quality of life studies where seeking self-report from children can be compromised by age and comprehension issues.

### Child Health Utility – 9D

The CHU9D, described previously, consists of 9 items each with a 5-level response category. Each item taps into a different domain (worry, sadness, pain, tiredness, annoyance, school, sleep, daily routine and activities). The time frame for the questions is “today”. Because of this, we asked a sub-sample of participants an additional question of whether “today” was a typical day for the child, to determine the representativeness of the child’s functioning on that day, of their general functioning. In cases where participants struggled to rate their child’s behaviour on that day, we asked them to rate their child’s behaviour on an average day. In examining the performance of the CHU9D, we present utility weights using both available tariffs, the UK Adult Tariff and the Australian Adolescent Tariff. Completed CHU9D questionnaires were scored using SPSS syntax provided by the authors of the tariffs [10,17].

### Strengths and Difficulties Questionnaire

The Strengths and Difficulties Questionnaire (SDQ) [6] was first developed as a shorter alternative to behavioural screening questionnaires such as the Rutter [26] and Child Behaviour Checklist [27,28] but with an additional focus on young people’s “strengths”. The SDQ has repeatedly demonstrated equivalence to these longer measures in terms of factor structure, reliability, sensitivity to detecting psychiatric diagnoses, and sensitivity to change [29-31]. The instrument is now a widely-used mental health screening measure in children and adolescents aged 4–17 years. In fact the SDQ is now a mandated consumer self-report routine outcome measure in Australian CAMHS [32], and a standard measure in UK routine outcome collections [33].

The SDQ comprises 25 items, each describing a psychological or behavioural attribute (some positive, some negative) which the responder indicates as being “very true”, “somewhat true” or “not true” of the child/adolescent in question over the last 6 months. The instrument generates both a total score and scores for 5 subscales including emotional, conduct, hyperactivity-inattention, peer problems and prosocial behaviour. The total score ranges from 0–40 with higher values indicating greater behavioural and emotional pathology. Individual sub-scales are scored from 0–10 with higher scores indicating poorer functioning for four of the subscales (emotional, conduct, hyperactivity-inattention and peer problems), and better functioning for one of the subscales (prosocial). There are also cutoff scores available for the sub-scales and the total score that define the following clinical bands: normal, borderline and abnormal. These are based on a population-based UK survey in which cutoffs were chosen such that 80% of children scored normal, 10% scored borderline and 10% scored abnormal. The SDQ is available in 3 forms - adolescent self-report, caregiver-administered and teacher-administered. We used the caregiver-administered form in this study and utilised SPSS syntax from the SDQ website [34] to score the instrument. The impact supplement was not used in this study.

### Crossover between measures

Only the 3 emotionally-related items (worried, sad, annoyed) in the CHU9D have obvious corollaries in the SDQ (Table 2). Furthermore the 2 instruments ask about quite different reference periods: the CHU9D asks about the child’s functioning ‘today’ whilst the SDQ asks about the previous 6 months.

**Table 2:** **Mapping of CHU9D items to SDQ items**

**CHU9D items**	**Corresponding SDQ items**
How worried is your child today?	Many worries or often seems worried
	Nervous or clingy in new situations, easily loses confidence
	Many fears, easily scared
How sad is your child today?	Often unhappy, depressed or tearful
How much pain does your child have today?	NA
How tired is your child today?	NA
How annoyed is your child today?	Often loses temper
How is your child doing with their schoolwork/homework today?	NA
How did your child sleep last night?	NA
How is your child doing with their daily routine today?	NA
To what extent can your child join in with activities today?	NA

### Analysis

Analyses were conducted using SPSS Version 19 and according to the following procedure:

- Data was screened and cleaned. There was 1 missing CHU9D data point and 26 missing SDQ data points. Two SDQ items (“kind to younger children” and “steals”) were the most frequently missed and comprised 9 data points in total, whilst other missing data points were scattered across the remaining 23 items. Missing data represented just 0.4% of all data items. A review of the raw SDQ questionnaire data revealed caregivers commonly reported “don’t know” on these items. For analysis purposes, a “no problem” approach was taken where missing values for these data points were replaced with the equivalent value for no problem. For the 1 missing CHU9D item, the same “no problem” approach was taken.
- Descriptive statistics such as age and gender were tabulated.
- SDQ subscale and total scores were calculated using syntax available from the SDQ website [34].
- CHU9D raw scores were calculated and translated into utility weights using the original UK Adult Tariff [17] and the Australian Adolescent Tariff [10].
- CHU9D raw and utility weights were tabulated and divided by respondent characteristics.
- Research Question 1 (face validity and practicality) was addressed by comparing the proportion of missing items from the CHU9D with the SDQ. We also utilised qualitative information collected by interviewers during the study on which questions caused the most difficulties for respondents in answering.
- Question 2 (reliability) was addressed by calculating Cronbach’s alpha for the CHU9D. Given that the CHU9D items tap into the same overall construct (quality of life) but represent different domains, we set an alpha of 0.7 as a minimally acceptable level of internal consistency [35].
- Question 3 and 4 (convergent validity) were addressed as following:
- We calculated Pearson’s product moment correlations between the CHU9D utility weight (both tariffs) and the SDQ total score. We used Cohen’s [36] categorisations to describe the strength of the correlation (0.1 = small, 0.3 = moderate, 0.5 = large).
- We generated an item-level correlation matrix of CHU9D and SDQ items to look for correlations between items, particularly those that were conceptually related.
- We regressed the SDQ total score on the individual items of the CHU9D using simple linear regression with all CHU9D items entered simultaneously. We used R^2^ and adjusted R^2^ to determine the variance in SDQ scores explained by the CHU9D. We used significance on B values to determine which CHU9D items were most predictive of SDQ Total scores.
- CHU9D utility weights were tabulated by respondent characteristics; age, gender, SDQ clinical band.
- Mann-Whitney U and Kruskal-Wallis tests were used to test for differences between groups based on these respondent characteristics. Non-parametric tests were used because CHU9D utility weights were not normally distributed. A difference of ≥ .03 in utility scores was considered clinically significant based on Drummond’s [37] ‘rule of thumb’.
- Question 5 (validity of utility weights) was addressed by comparing the mean utility weights (both tariffs) from the current sample against mean utility weights from (a) other studies using the CHU9D, and (b) other utility studies of child and adolescent mental health populations.

## Results

A total of 900 participants met the inclusion criteria during the data collection period and were randomised for contact. Interviewers attempted to contact caregivers by moving sequentially through the list of caregivers until 200 interviews were completed. This resulted in 407 caregivers being approached, of whom 150 were not contactable, 37 declined to be interviewed, 14 were discovered not to meet the criteria and 6 interviews were not completed. Descriptive statistics for the full sample (missing data imputed) are presented in Table 3.

**Table 3:** **Descriptive characteristics of participating caregivers and their children**

	**Participants (N = 200)**
Age of child – Mean (SD)	11.71 (5.75)
Age band, %	
5-7	10.5
8-10	25
11-13	31
14-17	33.5
Gender of Child, %	
Male	52.5
Female	47.5
Caregiver, %	
Mother	87
Father	8.5
Other	4.5
First time with CAMHS, %	
Yes	74.5
No	25.5
Length of time with CAMHS for current Episode (months), mean (SD)	11.95 (16.6)
Strengths and Difficulties Questionnaire (SDQ)	
Total score, mean (SD)	19.52 (7.87)
Emotion Subscale, mean (SD)	5.40 (2.55)
Conduct Subscale, mean (SD)	4.01 (2.86)
Hyperactivity Subscale, mean (SD)	6.41 (2.81)
Peer Subscale, mean (SD)	3.71 (2.43)
Prosocial Subscale, mean (SD)	6.92 (2.23)
SDQ Clinical Band, band (%)	
Normal	22
Borderline	12
Abnormal	66
CHU9D Raw score, mean (SD)	18.51 (6.64)
CHU9D Raw score, median (IQR)	17.00 (13–23)
CHU-9D Utility score	
UK Adult Tariff, mean (SD)	0.803 (.117)
Australian Adolescent Tariff, mean (SD)	0.739 (.145)

Three-quarters of participants were first-time CAMHS clients. Most (87%) participants were mothers. Based on SDQ scoring guidelines for total problems, 132 of the children were in the clinical range (66%), 24 were in the borderline range (12%) and 44 were in the normal range (22%). The proportion of children with scores indicating clinically significant problems in specific domains were as follows: emotional problems (60%), conduct problems (51%), hyperactivity (51%), peer problems (50%) and prosocial (17%). Two-thirds of children had difficulties in 2 or more areas and almost 30% of children had difficulties in 4 or more areas.

Consistent with it being a clinical sample, the mean SDQ total score was in the clinical range, and emotion, conduct, hyperactivity and peer subscales were all considerably higher (i.e. worse) than published Australian norms collected from parents of children in a similar age bracket (7–17) [38].

**Figure 1:**
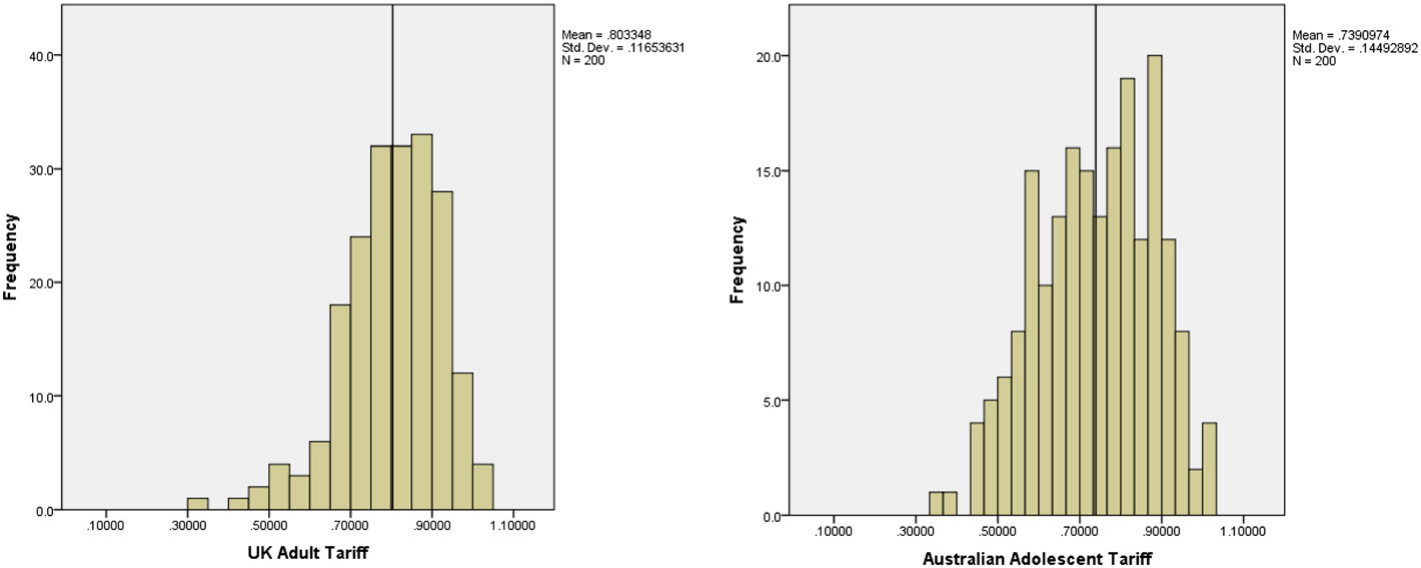
**Distribution of CHU9D utility weights for UK and Adolescent Tariffs.**

Weights from the UK Adult Tariff (Mdn = .819) were significantly higher than those from the Australian Adolescent Tariff (Mdn = .746), T = 743, p = .000. In fact, 87% of participants had a higher utility weight when using the UK Adult Tariff compared to the Australian Adolescent Tariff. The distributions of utility weights from the 2 tariffs are shown in Figure 1.

### Question 1 – face validity and practicality

Interviewers reported that the CHU9D proxy was simple and quick to administer, typically taking less than 2 minutes to complete. There was only one missing CHU9D data point across the 200 participants, indicating the CHU9D was well suited to interviewer administration to proxies.

Ninety (90) participants were asked if “today” (the reference time frame for the CHU9D) represented a typical day in terms of the child’s behaviour. This question was added to the survey after some parents reported the child’s behaviour during the survey period to not be representative of their general behaviour. Twenty-nine (32%) reported ‘no’ suggesting 1/3 of CHU9D ratings might not accurately capture the child’s average level of functioning. Of these 29, 18 (62%) indicated that today was ‘better than usual’ indicating a very subtle bias at the group level for the caregiver-completed CHU9D to underestimate dysfunction in some children.

Another issue encountered by interviewers was that 3 parents struggled to answer the typical day question because of limited exposure to their child that day and hence lack of knowledge about their mood, sleep, school and daily routine.

### Question 2 – internal consistency

The Cronbach alpha for the CHU9D was .781, indicating an acceptable level of internal consistency.

### Questions 3 and 4 – convergent validity

**Figure 2:**
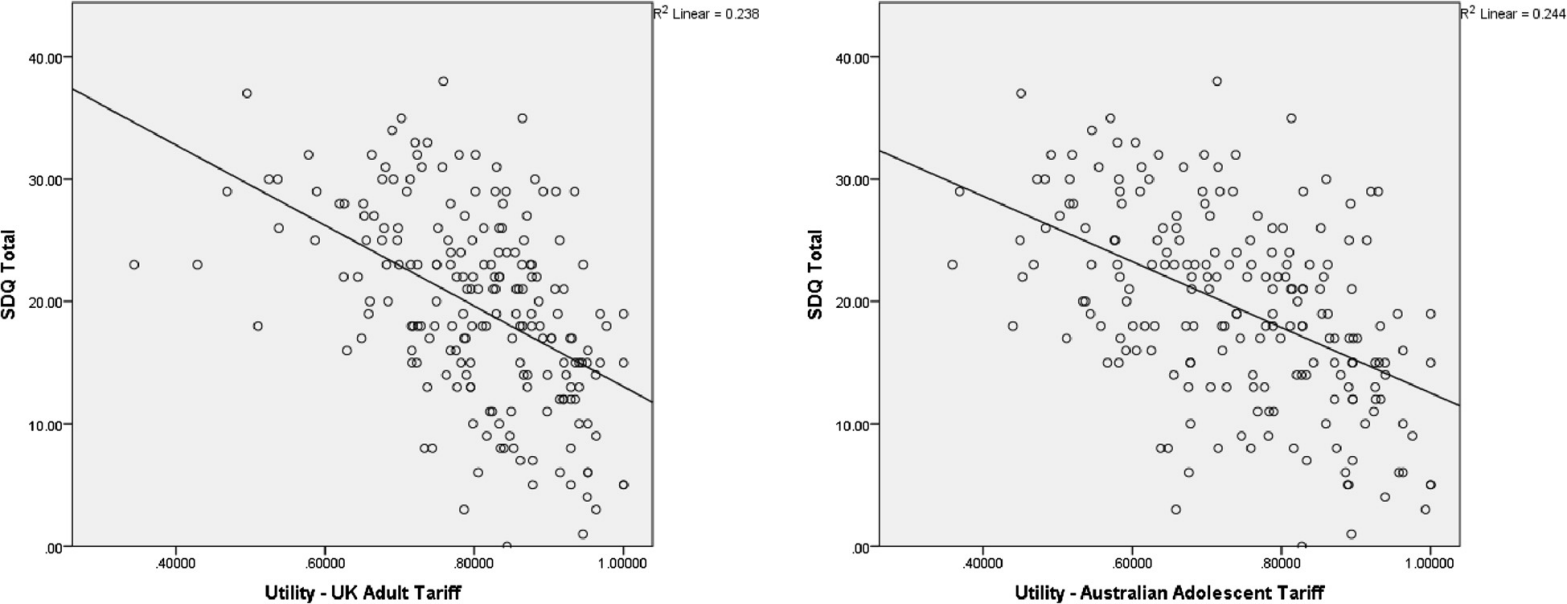
**Scatterplot of CHU9D utility weights and SDQ scores by Tariff.**

Figure 2 shows the scatterplot of CHU9D utility values (UK Adult and Australian Adolescent tariffs) and SDQ Total scores. Utility weights were moderately correlated with the SDQ total score, for both the UK Adult Tariff [r(199) = −.487 (p < .001)] and Australian Adolescent Tariff [r(199) = −.494 (p < .001)].

The correlation matrix of CHU9D and SDQ items (Table 4) revealed a predictable pattern. The strongest correlations were between the utility weights and the SDQ total score. The strongest item-level correlations were generally between conceptually overlapping items (e.g., ‘*many worries’* on SDQ and ‘*worried*’ on CHU9D, or ‘*often unhappy’* on SDQ and ‘*sad’* on CHU9D). The directions of the correlations were all in expected directions.

**Table 4:** **Correlation matrix of CHU9D and SDQ items**

	**CHU9D worried**	**CHU9D sad**	**CHU9D pain**	**CHU9D tired**	**CHU9D annoyed**	**CHU9D schoolwork**	**CHU9D sleep**	**CHU9D daily routine**	**CHU9D activities**	**Utility UK adult tariff**	**Utility Australian adolescent tariff**
**SDQ considerate**	.043	.007	.054	-.059	-.170*	-.233**	-.031	-.190**	-.169*	.151*	.119
**SDQ restless**	.089	.148*	.040	.113	.267**	.140*	.144*	.181*	.096	-.242**	-.242**
**SDQ sickness**	.191**	.182**	.196**	.260**	.169*	.108	.275**	.214**	.178*	-.314**	-.315**
**SDQ shares**	-.066	-.055	-.066	-.043	-.081	-.144*	-.155*	-.037	-.161*	.136	.121
**SDQ loses temper**	.204**	.180^*^	-.064	.180*	.324**	.290**	.188**	.248**	.178*	-.327**	-.342**
**SDQ pref solitary**	.018	.183**	.063	.084	.125	.217**	.209**	.201**	.168*	-.233**	-.207**
**SDQ well behaved**	-.041	-.146*	.148*	-.138	-.261**	-.294**	-.263**	-.338**	-.292**	.332**	.317**
**SDQ many worries**	.414**	.292**	.260**	.209**	.309**	.033	.269**	.012	.076	-.321**	-.364**
**SDQ help others if hurt**	-.014	-.026	.052	-.007	-.111	-.120	-.022	-.186**	-.279**	.173*	.116
**SDQ fidgeting**	.064	.173*	.019	.065	.226**	.159*	.169*	.218**	.174*	-.255**	-.242**
**SDQ one good friend**	-.112	-.121	.008	-.087	-.232**	-.060	-.216**	-.221**	-.216**	.254**	.261**
**SDQ often fights or bullies**	.076	.051	.007	.117	.298**	.219**	.095	.151*	.099	-.227**	-.218**
**SDQ often unhappy**	.217**	.334**	.076	.107	.305**	.079	.293**	.061	.184**	-.296**	-.318**
**SDQ generally liked by others**	-.077	-.070	.087	-.100	-.153*	-.176*	-.071	-.239**	-.197**	.187**	.167*
**SDQ easily distracted**	.134	.148*	-.074	.130	.235**	.322**	.135	.220**	.247**	-.290**	-.264**
**SDQ low confidence**	.183**	.156*	.104	-.010	.143*	.054	.054	.020	.096	-.129	-.125
**SDQ kind to younger children**	.000	-.047	-.003	-.075	-.188**	-.148*	-.104	-.138	-.144*	.191**	.162*
**SDQ often lies/cheats**	.076	.151*	-.031	.123	.282**	.272**	.193**	.188**	.230**	-.286**	-.272**
**SDQ picked on or bullied**	.170*	.198**	.085	.127	.231**	.005	.167*	.099	.165*	-.252**	-.257**
**SDQ volunteers to help**	.000	-.053	-.069	-.035	-.062	-.233**	-.009	-.127	-.219**	.163*	.124
**SDQ thinks before acting**	-.045	-.067	.126	-.106	-.193**	-.291**	-.155*	-.217**	-.252**	.228**	.196**
**SDQ steals**	.016	.108	-.034	.113	.144*	.234**	.152*	.341**	.171*	-.247**	-.223**
**SDQ better with adults than peers**	.217**	.105	.091	.114	.118	.128	.198**	.028	.216**	-.211**	-.215**
**SDQ many fears**	.239**	.162*	.105	.143*	.221**	.120	.278**	.083	.113	-.224**	-.259**
**SDQ good attention span/follow through**	.014	-.041	.083	-.077	-.109	-.436**	-.084	-.243**	-.126	.230**	.171*
**SDQ Total**	.250**	.305**	.049	.235**	.424**	.368**	.351**	.350**	.352**	-.506**	-.497**

**correlation is significant at the .01 level (two-tailed).

*correlation is significant at the .05 level (two-tailed).

Bolded figures are those where CHU9D and SDQ items have clear conceptual overlap.

A linear regression predicting the SDQ total score by CHU9D items (Table 5) revealed that the 9 CHU9D items explained 31.5% of the variance in SDQ total scores. Four items: annoyed, schoolwork, sleeping and daily routine, emerged as significant predictors.

**Table 5:** **Linear regression predicting SDQ total score from CHU9D items**

	***B (95% CI)***	***SE B***	**β**	**p**
Constant	8.22 (5.35, 11.094)	1.46		.000
CHU9D worried	.668 (−.213, 1.548)	.446	.109	.136
CHU9D sad	-.103 (−1.18, .973)	.545	-.015	.851
CHU9D pain	-.385 (−1.44, .673)	.537	-.047	.474
CHU9D tired	-.476 (−1.27, .313)	.400	-.083	.236
**CHU9D annoyed**	**1.355 (.472, 2.24)**	.**448**	.**238**	.**003**
**CHU9D schoolwork**	**.943 (.249, 1.64)**	.**352**	.**174**	.**008**
**CHU9D sleep**	**1.533 (.651, 2.41)**	.**447**	.**232**	.**001**
**CHU9D daily routine**	**1.194 (.136, 2.25)**	.**537**	.**155**	.**027**
CHU9D activities	.766 (−.218, 1.75)	.499	.107	.126

Note: R^2^ = .346, Adjusted R^2^ = .315.

Table 6 summarises mean (SD) and median (IQR) CHU9D weights according to respondent characteristics. Utility weights did not differ between age bands, or based on gender or first time with CAMHS. Utility weights did however decrease linearly with increasing severity of the SDQ, thus demonstrating convergent validity. Post hoc tests revealed those in the abnormal band had significantly lower utility weights than those in the borderline and normal bands.

**Table 6:** **CHU9D weights by respondent characteristics**

**Characteristics**	**N (%)**	**CHU9D utility UK adult tariff: mean (SD)**	**CHU9D utility adolescent tariff: Mean (SD)**	**CHU9D utility UK adult tariff: median (IQR)**		**CHU9D utility Australian adolescent tariff: median (IQR)**	
**Total group**	200 (100)	.803 (.117)	.739 (.145)	.819 (.731-.886)		.746 (.631-.860)	
**Age – 5-12**	118 (59)	.813 (.108)	.744 (.143)	.828 (.743-.894)	U = −1.021, P = .307	.740 (.646-.862)	U = −.596, P = .307
**Age – 13-18**	82 (41)	.790 (.127)	.731 (.148)	.812 (.716-.875)		.752 (.604-.856)	
**Gender male**	105 (52.5)	.805 (.113)	.746 (.137)	.825 (.748-.878)	U = −.352, P = .725	.762 (.645-.855)	U = −.669, P = .503
**Gender female**	95 (47.5)	.801 (.121)	.731 (.153)	.801 (.716-.898)		.740 (.592-.862)	
**SDQ Clinical Band**							
**Normal**	44 (22%)	.877 (.072)	.839 (.106)	.878 (.821-.939)	H(2) = 35.50, P = .000	.872 (.760-.925)	H(2) = 35.84, P = .000
**Borderline**	24 (12%)	.852 (.102)	.793 (.139)	.869 (.770-.943)		.830 (.661-.918)	
**Abnormal**	132 (66%)	.770 (.117)	.696 (.138)	.787 (.698-.859)		.702 (.584-.810)	
**First time with CAMHS**							
**Yes**	149 (74.5)	.807 (.120)	.743 (.149)	.822 (.729-.901)	U = −1.01, P = .311	.760 (.624-.872)	U = −.817, P = .414
**No**	51 (25.5)	.793 (.107)	.727 (.134)	.813 (.733-.861)		.715 (.644-.822)	

### Question 5 – comparisons of utility weights

Mean utility weights were lower in this study than for 2 Australian community samples (aged 11–17) tested using the CHU9D self-report version [22,24]. Furthermore, utility weights in this sample were lower than the only published utility norms for this age range (0.90 to .92 — Canadian norms using Health Utilities Index [39]) consistent with the sample being taken from a clinical population.

There were few studies of mental health populations against which to compare the values obtained in this study. For the few studies available [40-43], mean utility values were considerably higher in our study (e.g. .739 and .803 compared with .468, .433, .432, .656 and .49). Comparisons between studies should be treated with caution however as different instruments were utilised (HUI3 and EQ-5D) and the populations are not necessarily comparable. Details of the comparisons are summarised in Table 7.

**Table 7:** **Comparison of utility weights from the current study with other mental health populations**

***Reference***	***Population***	***Instrument***	***Utility***
Current study	200 5–17 year olds with varied mental health presentations	CHU9D parent completed – UK adult tariff	.803 (.117)
	200 5–17 year olds with varied mental health presentations	CHU9D parent completed – Australian adolescent tariff	.739 (.145)
**CHU9D Studies**			
Stevens and Ratcliffe 2012 [9,10]	Community sample of 961 11 to 17 year olds	CHU9D self-report, web survey – UK tariff	.85
	Sub-sample of 67 with long-standing disability, illness or medical condition	CHU9D self-report, web survey – UK tariff	.80
	Sub-sample of 281 with score on KIDSCREEN below the median	CHU9D self-report, web survey – UK tariff	.789
Ratcliffe et al. 2012 [24]	Community sample of 710 11 to 17 year olds	CHU9D self-report, web survey –UK tariff	.931
**Other studies of mental health populations**			
Petrou & Kupek 2009 [41]	46 children (mean age 10.9) on a disability registry with behaviour disorders	HUI3 – caregiver completed	.468
	105 children (mean age 11) on a disability registry with autism spectrum disorder	HUI3 – caregiver completed	.433
	50 children (mean age 10.9) on a disability registry with hyperactivity disorders	HUI3 – caregiver completed	.432
Petrou et al. 2010 [40]	39 children (aged 11) from a longitudinal study of pre-term infants	HUI3 – caregiver completed	.656
Goodyer et al. 2008 [42]	200 11–17 year olds with depression prior to SSRI therapy	EQ-5D	.49
Bodden et al. 2008 [43]	59 children with anxiety disorders prior to receiving individual CBT	EQ-5D	.87
	57 children with anxiety disorders prior to receiving family CBT	EQ-5D	.83

## Discussion

Health economic evaluations are routinely using the QALY as a summary measure of health outcome. The value of the QALY is that it is a generic measure that enables comparisons between a diverse range of health services, programs and interventions. In a health system where budgets are limited but demand for health services is high, policy makers need to make decisions about what services, programs and interventions to fund. Consideration of cost per QALY is a key part of this decision making process [44].

In this context psychotherapy-based services such as specialist CAMHS are competing for resources against pharmaceutical companies who have developed medications for many of the conditions seen in CAMHS (e.g. methylphenidate for ADHD, antidepressants for depression and anxiety) and are seeking to have those medications included in pharmaceutical benefits schemes. Whilst pharmaceutical companies are well versed in the utilisation of PBHRQOL instruments and calculation of cost per QALY; such information is provided in guidelines for submissions to the Pharmaceutical Benefits Advisory Committee [45], there are relatively few cost-utility studies of psychotherapy interventions and the use of PBHRQOL instruments in CAMHS is rare. For example, a search on “utility” in the PEDE database [46] returned 173 cost-utility studies, of which only 12 were for child and adolescent mental health disorders and 9 of these were for pharmaceutical treatments. This state of affairs disadvantages psychotherapy-based CAMHS who lack QALY data to support the effectiveness of their interventions.

In this study we explored the potential value of the Child Health Utility (CHU9D) as a routine outcome measure for use in CAMHS. The CHU9D is a preference-based instrument that generates utility weights which can be used to calculate QALYs for use in health economic evaluations. Of particular interest was whether the CHU9D was quick and easy to use, whether it could act as a suitable proxy for mental health symptoms, and whether it generated utility weights similar to those measured in other child and adolescent mental health population studies.

From a clinical perspective, the CHU9D was quick and easy to administer, and caregivers had little trouble answering the questions, suggesting it could be implemented with minimal fuss for caregivers. Three of the instrument’s 9 items relate directly to emotional symptoms: worried, sad, and annoyed. Additionally, the 3 items that were found to be significant predictors of the SDQ total score — schoolwork, sleep, and daily routine — measure impacts in areas commonly disrupted in children with a wide variety of mental health disorders. In a recent study of clinician’s behaviour and attitudes towards routine outcome measurement, administrative load and instrument relevance were highlighted as barriers to implementation [47]. The brevity and broad clinical relevance of the CHU9D are therefore important when considering the likelihood of clinicians endorsing and taking up the instrument in clinical practice.

Two administration issues were identified and need to be explored further. The first is that the instrument asks about the child’s functioning ‘today’. Almost 1/3 of caregivers in our study reported that ‘today’ was not a typical day for the child which may have led some to underestimate their child’s dysfunction. The other finding was that a small number of parents were unable to rate the child’s functioning that day due to limited exposure to their child. In a population where it is common for separated parents to share access to their child, this issue may occur more frequently than in non mental health populations. We suggest one modification to the instrument which might help address this problem, subject to further testing. This is to adjust the wording for caregivers to rate a ‘typical day’ if they report not having the information required to answer for the actual day in question.

Psychometrically, the instrument performed adequately, although we were only able to test a couple of aspects of its performance. The obtained Cronbach alpha of .781 is challenging to interpret. On one hand, it compares favourably to the alpha of .66 found by Foster Page et al. [48], suggesting the items are converging better in a mental health population than in a dental population. It was also higher than our cutoff of 0.7 but not in high 0.9’s suggesting the items are tapping into a central construct (i.e. quality of life) without indicating some items are redundant. However, it is difficult to define an ideal value for alpha for an instrument that is designed to measure the multi-dimensional nature of quality of life in children. We expect that further validation exercises in clinical populations with samples large enough for factor analysis will help illuminate the factor structure of the instrument for different clinical populations.

In terms of validity, despite having a different focus and reference time period to the SDQ (today vs last 6-months), there was evidence of moderate convergence between the instruments. The correlation between the SDQ and the CHU9D was in the moderate to strong range, item-level correlations were in the expected direction, and children in the abnormal range on the SDQ showed significantly lower utility weights than children in the borderline/normal ranges. These findings are important as a predictable relationship between quality of life and child and adolescent mental health supports the use of PBHRQOL instruments in this population.

From an economic perspective, we noted two things in relation to the utility weights generated by the CHU9D tariffs. First, we noted utility weights from this study were significantly higher (i.e. indicating better quality of life) than those collected in other child and adolescent mental health populations. Whilst the comparison was highly fraught as the comparative studies used different instruments and populations, we believe this issue warrants further investigation. If competing instruments generate significantly different utility weights in the same population, the interpretation of economic evaluations may be influenced by choice of utility instrument in addition to the performance of assessed interventions, a finding noted elsewhere [49,50]. Our current hypothesis is that the CHU9D is overestimating quality of life compared to other instruments in mental health populations, consistent with that found in other CHU9D studies [24]. The implications for this in terms of choice of instrument to use in measuring the impact of child and adolescent mental health interventions needs further exploring.

Second, we noted the failure of both CHU9D tariffs to capture the full range of utility weights from 0–1. Both tariffs have a floor utility weight of .3, similar to that seen in the SF-6D, a widely used adult utility instrument [51]. This smaller range can lead to an over-prediction of utility in poor health states [52] and an underestimation of utility change in intervention studies [53]. Thus the CHU9D might over-predict utility in severe Child and Adolescent Mental Health Services presentations, for example, severe mental illness (schizophrenia, depression) with suicidal ideation and suicide attempts. Interventions evaluated using the instrument may also show smaller utility gains (higher cost-utility estimates), than might be seen in adult populations where the EQ-5D is used, which has a significantly wider score range. Both situations potentially disadvantage economic evaluations of interventions in child and adolescent mental health, compared to adult interventions. Within the limited utility range, the Australian Adolescent Tariff generated a wider spread of scores and consistently lower mean values. Thus we suggest there is still ongoing work with these tariffs, to reduce the floor effect and explore differences in ratings between adolescents and adults. We note for example that a recent modification of the classification system of the SF-6D has provided preliminary evidence of being able to reduce the floor effect [54], and such an approach may be relevant to the CHU9D.

### Limitations

Due to the nature of the study we were unable to test a number of useful metrics. For example, we were not able to calculate test-retest reliability or the sensitivity of the instrument to change in mental health symptoms over time, having only one measurement point. These are particularly important metrics, as the value of the CHU9D for economic evaluation will depend on its capacity to reliably detect change due to intervention, rather than large natural fluctuations in an individual’s responses. We were also unable to explore concordance between caregiver and child self-completed versions of CHU9D as the telephone survey methodology was not well suited to collection of responses from children and adolescents. As a result, we recommend further psychometric validation of the CHU9D with a focus on repeated measures and multiple raters (e.g. child, caregiver, therapist).

In terms of the study sample, although it was drawn randomly from a list of active CAMHS clients, difficulties in contacting people from the list possibly led to the sample being higher functioning than a true random sample. In essence, families who were not contactable were assumed to have greater dysfunction, although we did not have data to test this hypothesis. There is also the question of whether the study sample is representative of CAMHS clients elsewhere. Comparisons not reported in this paper however show SDQ scores in our sample compare very similarly to SDQ scores collected routinely from other Australian CAMHS, hence we have reasonable confidence that the study sample is at least fairly representative of clients of CAMHS in Australia.

It should also be noted that there is a broader debate relating to the use of preference-based quality of life instruments in children and adolescents [55]. Concerns include that the valuation procedures used in defining the tariffs may not translate well from adult measures to children and adolescents, the capacity of children to understand and complete instruments, the accuracy of proxy raters, the need to consider family interactions in children’s measures, and the wide variation in utility weights noted for different childhood disorders from existing instruments. We also note (and this applies to adult instruments as well) that tariffs generated in one population or age group may not be comparable to those in other populations, further hampering efforts to use preference-based HRQOL instruments to facilitate cross comparisons. Whilst it is beyond the scope of this paper to address these issues in any depth, it should be noted that there are valid arguments that preference-based instruments (as they currently stand) might not be the best fit for child and adolescent populations, and therefore alternative metrics of outcomes with economic relevance (e.g. school attendance) should be similarly explored.

### Future work

The telephone survey method used in this study proved to be a viable and efficient way of communicating with caregivers about the mental health and quality of life of their children receiving mental health services. The process, which was separate from their clinical care, caused minimal disruption to clients and therapists. As such, this method might be suitable for exploring test-retest, sensitivity to change, and comparisons between PBHRQOL instruments. Telephone or web-based survey methods may also be suitable for tracking adolescents receiving services. For example, Ratcliffe and colleagues were able to obtain consent and collect data from adolescents using a web-based survey [24]. Eliciting answers to the CHU9D from very young children has required direct contact [21], thus studies involving children as young as 5 might need to be situated within clinics.

Future studies could thus employ telephone and web-based survey methods, but with a larger range of utility measures, as well as a follow-up assessment and sub-samples with repeated measures. Studies looking to obtain scores from children may need to supplement telephone and web-based methods with assessments conducted within the clinical services themselves. Templates for such studies include the Multi Instrument Comparison Project [56]. The outcomes of comparing the performance of different utility instruments in children and adolescents would provide much clearer guidance on whether preference-based instruments are a suitable addition to mental health services, and if so, which ones are superior.

## Conclusion

In this preliminary exploration of the value of the CHU9D as a routine outcome measure in child and adolescent mental health services, we demonstrated clinical relevance, ease of use, and adequate psychometric performance. The results of this study show however that further validation is required, including how the instrument performs in evaluating change over time and developing tariffs to ensure the utility weights capture the full range of functioning observed in this population. Exploring and evaluating the use of preference-based health related quality of life utility instruments in CAMHS remains a priority, as use of such instruments will be essential for CAMHS to demonstrate effectiveness and economic salience as a health provider, and therefore allow such services to compete successfully for resources, in this climate of budgetary restraint.

## Abbreviations

CAMHS: Child and adolescent mental health services
CHU9D: Child health utility – 9 dimension
PBHRQOL: Preference-based health related quality of life
SDQ: Strengths and difficulties questionnaire
QALY: Quality adjusted life year
